# The effect of earthquake on fibromyalgia: a comparison of patients on medication and without medication

**DOI:** 10.1007/s00296-024-05605-5

**Published:** 2024-05-09

**Authors:** Gulsah Yasa Ozturk, Neval Bozok Arat, Asena Ayca Ozdemir, Ibrahim Bashan, Burhan Fatih Kocyigit

**Affiliations:** 1 Department of Physical Medicine and Rehabilitation, University of Health Sciences, Adana City Research and Training Hospital, Adana, Türkiye; 2 Department of Medical Education, Faculty of Medicine, Mersin University, Mersin, Türkiye

**Keywords:** Fibromyalgia, Earthquakes, Disasters, Psychological stresses, Emotional stress

## Abstract

**Introduction / objectives:**

Stressful events like earthquakes might worsen the symptoms of fibromyalgia, although the influence of medications on these consequences is yet uncertain. The objective of this study was to examine the influence of an earthquake on the symptoms of fibromyalgia and evaluate the impacts of medications used to treat fibromyalgia on the clinical picture.

**Method:**

Ninety-five fibromyalgia patients were enrolled in a comparative study and divided into two groups: medication and non-medication. Three subcategories of medication groups were established: selective serotonin reuptake inhibitors (SSRIs), serotonin-norepinephrine reuptake inhibitors (SNRIs), and gabapentinoid drugs (GDs). Before and after the earthquake, clinical evaluations were conducted using the Fibromyalgia Impact Questionnaire (FIQ), Hospital Anxiety and Depression Scale (HADS), and Jenkins Sleep Rating Scale (JSS). Statistical analyses were conducted to compare the scores before and after the earthquake and evaluate the differences between the groups.

**Results:**

Statistically significant increases were observed in FIQ, HADS-anxiety, HADS-depression, and JSS scores in the medication and non-medication groups before and after the earthquake comparisons (*p* < 0.05). Non-medication group reported significantly higher post-earthquake changes in FIQ, HADS-anxiety, HADS-depression, and JSS compared to the medication group (*p* < 0.05). While HADS-anxiety, HADS-depression, and JSS changes after the earthquake differed according to the drug subgroups (*p* < 0.05), no statistically significant difference was observed in FIQ values (*p* > 0.05). The highest scores were detected in the GD subgroup.

**Conclusions:**

This study highlights the substantial impact of earthquakes on fibromyalgia patients. Medication use may assist in reducing the detrimental effects of stresses like earthquakes on fibromyalgia symptomatology. Future research with larger sample sizes and more extended follow-up periods is needed to explain these findings and optimize treatment regimens for fibromyalgia patients experiencing significant stressors.

**Supplementary Information:**

The online version contains supplementary material available at 10.1007/s00296-024-05605-5.

## Introduction

Fibromyalgia is a condition characterized by broad symptomatology involving chronic widespread pain, fatigue, sleep disturbances, and headaches [1, 2]. Psychological and emotional situations are among the factors that influence and exacerbate fibromyalgia [3]. Tiredness, fatigue, depression, anxiety, reluctance, and boredom are more common in fibromyalgia patients [4]. Additionally, stress may increase fibromyalgia symptoms or contribute to the onset of the disease [5].

Earthquakes can cause emotional reactions, such as stress, anxiety, fear, and phobia [6]. Emotional responses can initiate or worsen the symptoms associated with chronic pain conditions like fibromyalgia. In particular, there is evidence that stress has a negative effect on fibromyalgia-related symptoms [5, 6].

The management of fibromyalgia typically necessitates a comprehensive strategy including multiple disciplines and should be customized to address the specific symptoms and requirements of each individual [7]. Depending on the clinician’s preference and the patient’s compliance, pharmacological agents such as tricyclic antidepressants, serotonin reuptake inhibitors (SSRIs), serotonin-norepinephrine reuptake inhibitors (SNRIs), and gabapentinoid drugs (GDs) are used in the treatment [8, 9].

The earthquake of 7.8 magnitude centered in Pazarcık district of Kahramanmaraş province of Turkey on February 6, 2023, affected ten provinces and caused over 40,000 casualties, over 100,000 injuries, and the evacuation of nearly 2,000,000 earthquake victims [10]. It is an undeniable fact that such a major disaster caused both individual and social trauma as well as loss of life and property [11, 12].

This study aimed to examine the impact of a significant stressor, such as an earthquake, on the clinical presentation of fibromyalgia and assess the impact of fibromyalgia-related medication use on the effects caused by an earthquake.

## Materials and methods

### Study design

This comparative study was conducted in a tertiary hospital’s physical medicine and rehabilitation department. To determine the difference in changes before and after an earthquake in fibromyalgia patients who use and do not use fibromyalgia-related drugs, with a medium (0.25) effect size, it is appropriate to include at least 60 participants in the study, with 80% power and a 5% types I error. The G*Power program was used for the calculation. A total of 95 patients aged 18 years and older with a previous diagnosis of fibromyalgia were included. The participants fulfilled the 2016 American College of Rheumatology criteria [13]. Exclusion criteria were inflammatory rheumatic diseases, autoimmune diseases, degenerative joint diseases, polyneuropathy, a disorder that may cause neurological deficit (multiple sclerosis, Parkinson’s disease and amyotrophic lateral sclerosis etc.), dementia, endocrine disorders (diabetes mellitus and thyroid dysfunction etc.), metabolic disorders, major depression or other psychiatric disorders (anxiety disorder and panic disorder etc.), paraneoplastic syndrome, medication use with the potential to affect scores, and a history of earthquake-related fracture, surgery, neuropathy or amputation. All subjects underwent routine blood tests. Subjects who did not fit the study criteria were dropped from the study. In addition, the hospital registry system was inspected for exclusion criteria. Data was collected for each subject, involving age, body mass index (BMI), gender, education, and occupation status. The participants were asked about their medication usage. The Ministry of Health system was used to verify prescription drugs. The duration of symptoms in individuals with fibromyalgia was also assessed.

The study consisted of two distinct groups: those who were regularly taking medicine for fibromyalgia and those who had not taken any fibromyalgia-related medication over the past six months. Three subgroups were formed from the medication group: selective serotonin reuptake inhibitors (SSRIs), serotonin-norepinephrine reuptake inhibitors (SNRIs), and gabapentinoid drugs (GDs) subgroups. Two clinical assessments were conducted, three months before and one month after the earthquake. Ethical approval of the study was obtained from local ethics committee with decision number 2523 − 125.

#### The fibromyalgia impact questionnaire

The Fibromyalgia Impact Questionnaire (FIQ) is a measurement tool initially created by Burckhardt et al. [14]. Sarmer et al. [15] completed its Turkish validity and reliability assessment. The FIQ’s objective is to evaluate the impact of fibromyalgia. The assessment includes data on the patient’s physical functioning, fatigue, energy levels, pain, sleep disturbances, and mental state. The questionnaire has ten primary inquiries. The highest possible score is 100, which indicates a more serious clinical condition as values increase.

#### Hospital anxiety and depression scale

The Hospital Anxiety and Depression Scale (HADS) was initially created by Zigmond and Snaith in 1983 and then adapted for use in Turkish by Aydemir et al. [16, 17]. It is commonly employed in medical environments, particularly in hospitals and clinics, to evaluate the psychological condition of individuals. The HADS consists of two subscales that evaluate both anxiety and depression. The HADS typically consists of 14 items, divided equally into seven questions for the anxiety subscale and seven for the depression subscale. Each question pertains to a distinct emotional condition and allows patients to assess the emotional states they have encountered during a specified timeframe (usually within the last week).

#### Jenkins sleep evaluation scale

Jenkins et al. [18] established the Jenkins Sleep Rating Scale (JSS), a measurement instrument for evaluating sleep disturbances. Duruöz et al. [19] conducted a Turkish validity and reliability study. The questionnaire assessed issues such as “difficulty falling asleep, waking up several times during the night, not being able to sleep without waking up during the night, feeling tired and exhausted after waking up during a normal sleep period.”

These assessment parameters are the scales we routinely record in managing fibromyalgia patients in our clinic. One month after the earthquake, patients with follow-up were invited to our clinic for re-evaluation. The data three months before the earthquake were taken as the basis for the pre-earthquake data. Although three months before the earthquake was determined as a point, two weeks before and two weeks after this point were also accepted.

### Statistical analysis

The Shapiro-Wilk test evaluated normality control of continuous variables. Parametric methods were used in the analysis of variables that conformed to the normal distribution, and nonparametric methods were used in those that did not, and descriptive statistics were expressed as mean, standard deviation (SD), median, interquartile range (IQR), minimum, and maximum values. Independent sample t-test and Mann Whitney U test were used to compare patients using and not using a medication, while paired t-test and Wilcoxon test were used to compare pre-and post-earthquake values. The difference between pre-and post-earthquake changes according to drug use groups was analyzed by repeated measures ANOVA. Kruskal Wallis test was used for the comparison according to drug groups. Fisher Exact and Chi-Squared tests were used for categorical variables, and descriptive statistics were expressed as frequency and percentage. Data analysis was evaluated in Statistica® 13.5.0.17 program. “Relative Changes” is an expression of the % change in the post-earthquake change compared to the pre-earthquake values and was calculated as follows.

$$\begin{array}{l}Relative\,{\mkern 1mu} Changes = \\\frac{{(After\,Earthquake - Before\,Earthquake)}}{{Before\,Earthquake}}\\x\,100\end{array}$$

## Results

A total of 95 fibromyalgia patients were included in the study. While 40 (42.1%) of the patients were taking medication for fibromyalgia, 55 (57.9%) were not. The mean age of the patients was 50.07 ± 10.72 years, BMI was 28.20 ± 4.51, and median duration of fibromyalgia was 6 (min:3, max:10) years. Ninety (94.7%) of the patients were female, 53 (55.8%) were primary school graduates, 10 (10.5%) were secondary school graduates, 18 (18.9%) were high school graduates, 14 (14.7%) were university graduates, and 13 (13.7%) were working. When we compared the characteristics of the patients according to the use of medication, no significant difference was detected (*p* > 0.05) (Table 1).

**Table 1:** Baseline characteristics of fibromyalgia patients

	Non-medication users (*n* = 40)		Medication users (*n* = 55)		*p* ^1^
	Mean ± SD Median [IQR]	Min-Max	Mean ± SD Median [IQR]	Min-Max	
Age	47.55 ± 10.6	25–75	51.91 ± 10.52	28–70	0.050
BMI	28.37 ± 4.76	17.04–38.46	28.08 ± 4.37	19.38–37.25	0.765
Symptom duration (years)	6 [2–10]	1–16	6 [4–10]	1–30	0.631*
	***n***	**%**	***n***	**%**	***p*** ^**2**^
Gender					
Male	3	7.5	2	3.6	0.647
Female	37	92.5	53	96.4	
Education					
Primary School	20	50.0	33	60.0	0.539*
Middle School	6	15.0	4	7.3	
High School	7	17.5	11	20.0	
University	7	17.5	7	12.7	
Working					
No	34	85.0	48	87.3	0.750*
Yes	6	15.0	7	12.7	

*p*^1^:Independent sample t test, *Mann Whitney U test; p^2^:Fisher Exact test, *Chi-Square test

SD: Standard deviation, IQR: Interquartile range, Min: Minimum, Max: Maximum, n: Number; %: Percentile, BMI: Body mass index

In pre-earthquake assessments, HADS-anxiety level was significantly higher in the medication users group (*p* = 0.01). On the other hand, there was no significant difference between the groups regarding FIQ, HADS-depression, and JSS (*p* > 0.05). When the pre- and post-earthquake scores were compared within groups, both groups showed statistically significant increases in FIQ, HADS-anxiety, HADS-depression, and JSS scores (Table 2).

**Table 2:** Comparisons of FIQ, HADS and JSS scores of fibromyalgia patients before and after the earthquake according to medication status

	Non-medication users (*n* = 40)			Medication users (*n* = 55)			*p*_group_
	Mean ± SD	Median [IQR]	Min - Max	Mean ± SD	Median [IQR]	Min - Max	
**FIQ**							
Before	42.28 ± 11.95	41.64 [34.79–53.3]	22.4–67.15	45.85 ± 16.3	40.18 [34.15–52.94]	20.15–87.09	0.752
After	63.74 ± 13.54	61.2 [54.96–77.77]	37.41–88.9	50.6 ± 17.29	49.62 [36–65.91]	20.15–90.02	**<0.001**
Difference	20.47 ± 12.13	21.46 [10.81–25.99]	0–49.27	4.75 ± 11.47	0 [0–5.74]	-18.58–44.44	**<0.001** ^**ŧ**^
*p*_time_	**< 0.001**			**<0.001**			
**HADS (Anxiety)**							
Before	7 ± 3.55	7 [4–10]	0–15	9.15 ± 4.14	8 [6–12]	2–19	0.010*
After	12.4 ± 4.29	13 [10.25–15.75]	1–20	10.69 ± 4.2	11 [7–14]	2–21	**0.030**
Difference	5.4 ± 3.06	6 [3–7.75]	0–14	1.55 ± 2.14	1 [0–2]	0–8	**<0.001** ^**ŧ**^
*p*_time_	< 0.001*			**<0.001**			
**HADS (Depression)**							
Before	6.75 ± 3.4	6 [4–8.75]	0–14	8.29 ± 3.75	7 [5–10]	2–17	0.086
After	12.05 ± 4.5	14 [8.5–14.75]	2–18	9.82 ± 4.28	9 [6–13]	2–19	**0.014**
Difference	5.3 ± 3.2	6 [4–8]	0–12	1.53 ± 2.25	0 [0–3]	-2–8	**<0.001** ^**ŧ**^
*p*_time_	**< 0.001**			**<0.001**			
**JSS**							
Before	8.3 ± 5.22	10 [4–10.75]	0–20	10.44 ± 5.21	10 [7–14]	1–20	0.089
After	13.48 ± 5.1	14 [10.25–18]	2–20	11.78 ± 4.99	13 [8–15]	1–20	0.099
Difference	5.18 ± 3.05	6 [2–8]	0–9	1.35 ± 2.1	0 [0–2]	0–9	**<0.001** ^**ŧ**^
*p*_time_	**< 0.001**			**<0.001**			

*p*_group_: Mann Whitney U test *Independent Sample t test, p_time_:Wilcoxon test *Paired t test, ŧ:Repeated Measures ANOVA (timexgroup interactions)

FIQ: Fibromyalgia Impact Questionnaire, HADS: Hospital Anxiety and Depression Scale; JSS: Jenkins Sleep Evaluation Scale

SD: Standard deviation, Min: Minimum, Max: Maximum, n: Number

In post-earthquake evaluations, the non-medication group had significantly higher FIQ, HADS-anxiety, and HADS-depression scores (*p* < 0.05), but there was no significant difference in JSS scores (*p* = 0.09). In addition, the non-medication group showed significantly higher post-earthquake changes in FIQ, HADS-anxiety, HADS-depression, and JSS compared to the medication group (*p* < 0.05) (Table 2).

**Fig. 1:**
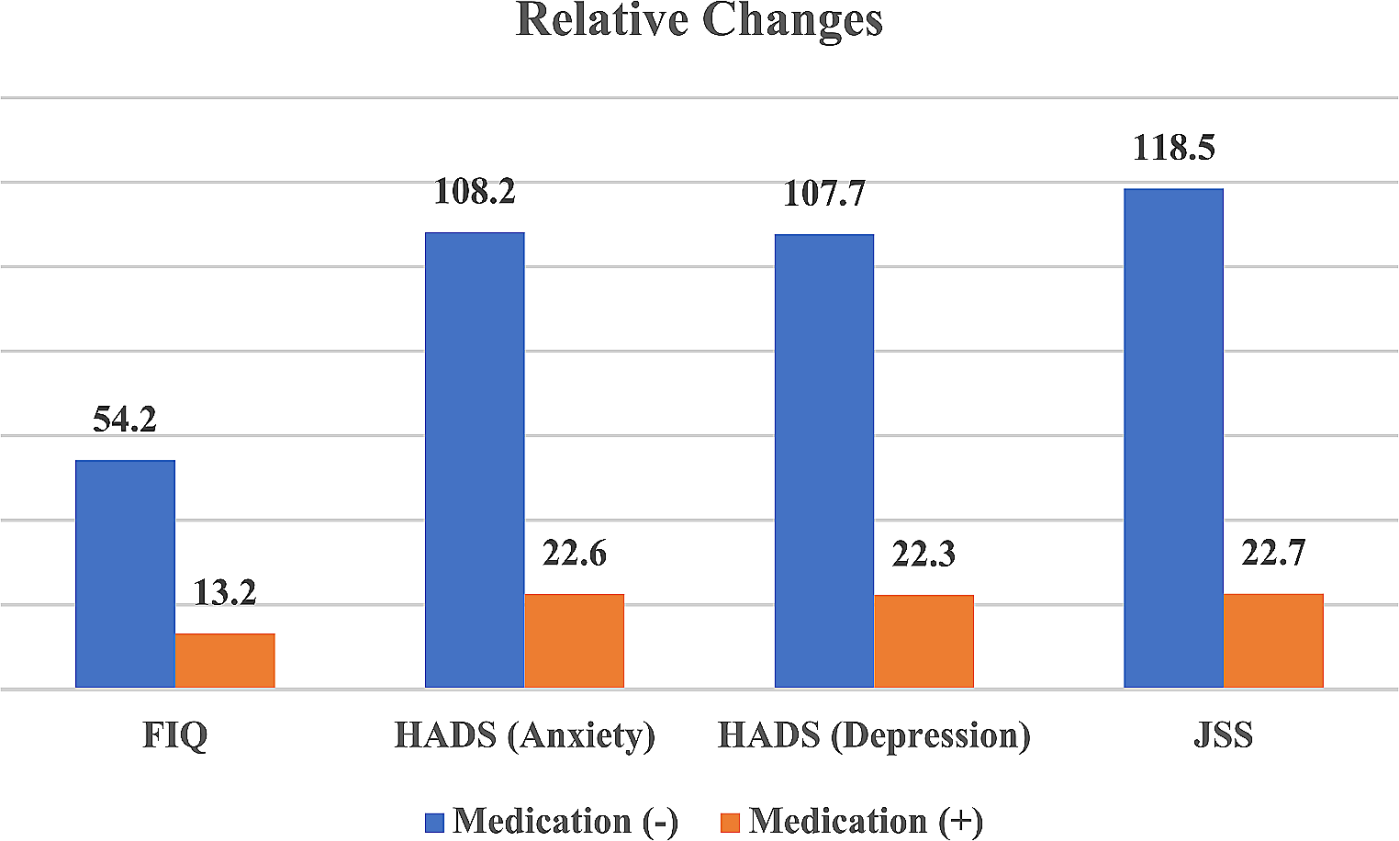
Relative change values of FIQ, HADS and JSS scores FIQ: Fibromyalgia Impact Questionnaire, HADS: Hospital Anxiety and Depression Scale; JSS: Jenkins Sleep Evaluation Scale

HADS-anxiety 108.2%, HADS-depression 107.7%, JSS 118.5%, and FIQ value 54.2% relative to the pre-earthquake level in patients who did not use medication, whereas, in the group who used medication, this was observed as 22.6%, 22.3%, 22.7%, and 13.2%, respectively (Fig. 1).

While HADS-anxiety, HADS-depression, and JSS changes after the earthquake differed according to the drug groups (*p* < 0.05), no statistically significant difference was observed in FIQ values (*p* > 0.05). Post-earthquake anxiety and depression scores increased more in GD users compared to SNRIs and SSRIs groups, and this calculated value was found to be statistically significant (*p* < 0.05). Post-earthquake JSS levels in GD users increased more than in SNRI users (*p* < 0.05). When the changes for FIQ were analyzed, the calculated values were not statistically significant (*p* > 0.05) (Table 3).

**Table 3:** Comparison of changes in FIQ, HADS and JSS scores after the earthquake according to the medication groups

	SNRIs (*n* = 26)			Gabapentinoid (*n* = 21)			SSRIs (*n* = 6)			*p*
	Mean ± SD	Median [IQR]	Min - Max	Mean ± SD	Median [IQR]	Min - Max	Mean ± SD	Median [IQR]	Min - Max	
FIQ	2.74 ± 9.1	0.01 [0-2.96]	-10.05–44,44	5.05 ± 11.52	1 [0–12.73]	-18.58–30.72	1.57 ± 3.85	0 [0-2.36]	0- 9.42	0.475
HADS (Anxiety)	0.54 ± 0.81	0 [0–1]	0–2	2.86 ± 2.39	3 [0.5–5]	0–7	0.17 ± 0.41	0 [0-0.25]	0–1	**< 0,001**
HADS (Depression)	0.31 ± 0.79	0 [0–1]	-2–2	3.1 ± 2.43	3 [0–5]	0–8	0 ± 0	0 [0–0]	0–0	**< 0,001**
JSS	0.38 ± 0.64	0 [0–1]	0–2	2.19 ± 2.4	2 [0–4,5]	0–6	1 ± 1.55	0.5 [0-1.75]	0–4	**0,047**

*p*: Kruskal Wallis Test, SNRIs: Serotonin-norepinephrine reuptake inhibitors, SSRIs: serotonin reuptake inhibitors

SD: Standard deviation, Min: Minimum, Max: Maximum, n: Number, IQR: Interquartile range

FIQ: Fibromyalgia Impact Questionnaire, HADS: Hospital Anxiety and Depression Scale; JSS: Jenkins Sleep Evaluation Scale

## Discussion

Our article demonstrates that FIQ, HADS-anxiety, HADS-depression, and JSS scores deteriorated in both the medication and non-medication groups during the initial month following the earthquake. Individuals who did not use medication displayed notably greater increases in FIQ, HADS-anxiety, HADS-depression, and JSS following the earthquake, in comparison to those who used medicine. When categorizing medicine users into three categories - SNRI, GD, and SSRI - it was determined that the GD group experienced the greatest impact.

Studies demonstrated that the clinical picture of individuals with fibromyalgia is impacted following an earthquake. Following the Japan earthquake, a rise in post-traumatic stress was noted in around 57% of patients with fibromyalgia [20]. Multiple studies indicated that earthquakes have detrimental impacts on mental health, depression, anxiety, and sleep [6, 21, 22]. A study conducted in Italy found that patients with fibromyalgia experienced an increase in their FIQ scores at 1, 2, 4, and 6 months following the earthquake [23]. Our results align with the data in the existing literature. An earthquake, acting as a significant trigger, heightened the disease activity of individuals with fibromyalgia and negatively impacted their levels of depression, anxiety, and sleep quality. Earthquakes are accompanied by multiple stressors that depend on individual issues (e.g. coping strategies), societal impact (e.g. personal and economic losses) and contextual issues (e.g. family network and financial problems). In this article, we consider the earthquake as the overarching factor of all these stressors [24]. The relationship between catastrophic events, such as an earthquake, and the manifestation of fibromyalgia symptoms can be intricate and differs from individual to individual. Stress is frequently mentioned as a catalyst for the onset or worsening of fibromyalgia symptoms. Individuals with fibromyalgia can have a notable deterioration in symptoms and encounter more challenges in controlling their condition as a result of earthquakes and other stressful events [25]. Healthcare practitioners must be cognizant of these potential consequences and offer suitable assistance and resources to aid clients in managing such demanding circumstances.

In this study, we divided the individuals into two groups: those who used medicine for fibromyalgia and those who did not. Following assessments conducted after the earthquake, the non-medication group had significantly higher scores in FIQ, HADS-anxiety, and HADS-depression. However, there was no notable difference in JSS scores. Furthermore, individuals who did not use medication exhibited more significant post-earthquake changes in FIQ, HADS-anxiety, HADS-depression, and JSS as compared to those who did use medication. Our findings indicate a notable impact linked to earthquakes in both medication and non-medication groups. Nevertheless, this impact is more profound in the non-medication group. There are numerous non-pharmacological treatment alternatives available for fibromyalgia, with exercise interventions being particularly prominent [26, 27]. Nevertheless, our findings indicate that in the event of severe stressors like an earthquake, medications used to treat fibromyalgia may somewhat mitigate the effects on patients. Hence, for fibromyalgia patients who are undergoing non-pharmacologic treatment, it may be advisable to transition to medication in the presence of a significant stressor.

We partitioned the medication group into three subgroups, SNRI, GD, and SSRI, and compared the scores. There was no significant difference in the FIQ score during the assessments conducted after the earthquake. However, there were significant discrepancies in the HADS-anxiety, HADS-depression, and JSS scores. The GD group obtained the highest scores. Our findings suggest SNRIs and SSRIs may be a more favorable choice in the presence of a substantial stressor, at least until that stressor is eliminated.

The paper has some limitations. The sample size is relatively small. Consequently, the number of participants in both the medication and non-medication groups is limited. The article relies on self-reported measures, which increases the potential for bias. There were no patients taking tricyclic antidepressant drugs. Finally, the duration of the follow-up period is restricted, and only evaluations conducted within the initial month after the earthquake are accessible. Hence, we are unable to provide any remarks regarding the long-term impacts of the earthquake on those who have fibromyalgia.

## Conclusion

This study highlights the substantial influence of earthquakes on patients with fibromyalgia, underlining that the disease severity, anxiety, depression, and sleep difficulties worsen following a traumatic event. Our research indicates that both medication and non-medication groups reported a deterioration in symptoms following the earthquake. However, the non-medication group experienced a considerably more pronounced increase in the severity of their symptoms. Furthermore, our analysis of fibromyalgia drug subgroups found that patients receiving SNRIs and SSRIs experienced lower increases in symptom severity than those taking GDs. This suggests that SNRIs and SSRIs may provide more effective symptom control in the face of significant stressors like earthquakes. Future study with larger sample sizes and more extended follow-up periods is needed to understand better the association between stressful events and fibromyalgia symptomatology, as well as the efficacy of various treatment modalities in mitigating these effects.

### Electronic supplementary material

Below is the link to the electronic supplementary material.

Supplementary Material 1

Supplementary Material 2

Supplementary Material 3

Supplementary Material 4

Supplementary Material 5

Supplementary Material 6

Supplementary Material 7

Supplementary Material 8

Supplementary Material 9

Supplementary Material 10
